# A Novel Non-Psychoactive Fatty Acid from a Marine Snail, *Conus inscriptus*, Signals Cannabinoid Receptor 1 (CB1) to Accumulate Apoptotic C16:0 and C18:0 Ceramides in Teratocarcinoma Cell Line PA1

**DOI:** 10.3390/molecules29081737

**Published:** 2024-04-11

**Authors:** Christina Sathyanathan Vijayaraghavan, Lakshmi Sundaram Raman, Shanmugapriya Surenderan, Harpreet Kaur, Mohanapriya Dandapani Chinambedu, Sadras Panchatcharam Thyagarajan, Mary Elizabeth Gnanambal Krishnan

**Affiliations:** 1 Department of Biotechnology, Faculty of Biomedical Sciences and Technology, SRI Ramachandra Institute of Higher Education and Research (SRIHER), Deemed to be University (DU), Porur, Chennai 600116, Tamil Nadu, India; christy.sathya@gmail.com; 2 Faculty of Pharmacy, SRI RAMACHANDRA Institute of Higher Education and Research (SRIHER), Deemed to be University (DU), Porur, Chennai 600116, Tamil Nadu, India; sundaram@sriramachandra.edu.in; 3 Whizbang Bioresearch Private Limited, Porur, Chennai 600077, Tamil Nadu, India; whizbangbioresearch@gmail.com; 4 Department of Human Genetics, Faculty of Biomedical Sciences and Technology, SRI Ramachandra Institute of Higher Education and Research (SRIHER), Deemed to be University (DU), Porur, Chennai 600116, Tamil Nadu, India; pre.singh@gmail.com (H.K.); mohanapriya@sriramachandra.edu.in (M.D.C.); 5 Distinguished Professor and Advisor to Chancellor, Vellore Institute of technology (VIT), Vellore Campus, Tiruvalam Rd, Katpadi, Vellore 632014, Tamil Nadu, India; profspt@gmail.com

**Keywords:** cannabinoid receptor 1 (CB1), FAAH1, ceramide, ovarian cancer, apoptosis, zebrafish models, MD simulation

## Abstract

The cannabinoid-type I (CB1) receptor functions as a double-edged sword to decide cell fate: apoptosis/survival. Elevated CB1 receptor expression is shown to cause acute ceramide accumulation to meet the energy requirements of fast-growing cancers. However, the flip side of continual CB1 activation is the initiation of a second ceramide peak that leads to cell death. In this study, we used ovarian cancer cells, PA1, which expressed CB1, which increased threefold when treated with a natural compound, bis(palmitoleic acid) ester of a glycerol (**C2**). This novel compound is isolated from a marine snail, *Conus inscriptus,* using hexane and the structural details are available in the public domain PubChem database (ID: 14275348). The compound induced two acute ceramide pools to cause G0/G1 arrest and killed cells by apoptosis. The compound increased intracellular ceramides (C:16 to 7 times and C:18 to 10 times), both of which are apoptotic inducers in response to CB1 signaling and thus the compound is a potent CB1 agonist. The compound is not genotoxic because it did not induce micronuclei formation in non-cancerous Chinese hamster ovarian (CHO) cells. Since the compound induced the cannabinoid pathway, we tested if there was a psychotropic effect in zebrafish models, however, it was evident that there were no observable neurobehavioral changes in the treatment groups. With the available data, we propose that this marine compound is safe to be used in non-cancerous cells as well as zebrafish. Thus, this anticancer compound is non-toxic and triggers the CB1 pathway without causing psychotropic effects.

## 1. Introduction

Several investigational approaches from the 1990s specify the fundamental roles played by cellular amounts of ceramide in regulating growth suppression, cell cycle arrest, apoptosis and/or cell senescence [1]. A huge spectrum of inducers of acute/de novo cellular ceramide synthesis were discovered in the last three decades for cancer-related therapies, which include but are not limited to stress-induced protein kinase activation, like Fas [2,3,4,5], CB1 [5,6] and CB2 [4]. In this context, natural and synthetic compounds, as well as endogenous cannabinoids that transduce molecular signals via CB1 and 2 subtypes, have been researched extensively in numerous cancers [7,8,9,10]. However, the only factor that limits the usage of CB ligands in cancer treatment is their inherent psychotropic property [11]. An array of compounds sourced from nature are used as cannabinoid modulators, notably curcumin [12], serinolamides [13] and betulinic acid [14], to state a few. These compounds are investigated as potent inhibitors of cancer progression, however, the psychoactive properties of many of these drugs have not been clarified. In line with this fact, attention is directed towards the exploration of non-psychoactive drugs that bind to CB1 or CB2, which are at the same time capable of killing cancer cells [15,16,17]. In this paper, we report, for the first time, a marine natural compound which is a non-psychoactive fatty acid, bis(palmitoleic acid) ester of a glycerol (**C2**), obtained from the deep water sea snail *Conus inscriptus* that possesses anticancer properties. We previously reported the effect of this compound on ovarian cancer cells and using computational analysis we indicated that the compound binds to the CB1 receptor at micromolar levels [18]. Alongside binding to CB1, treatment with **C2** also enhanced CB1 mRNA transcripts as well as protein expression in an ovarian cancer cell line, PA1. After extensive investigation, it was understood that this compound is a CB1 agonist and that **C2**-CB1 binding induced acute intracellular accumulation of C16:0-type ceramide threefold and C18:0 twofold within 24 h and killed the cells by apoptosis. Thus, the paper explores the possibilities of a novel natural compound to kill ovarian cancers by binding to the CB1 receptor, which at the same time is non-psychotropic so that it may be used in cancer therapeutics.

## 2. Results

### 2.1. Background Information on the Structural Details of the Compound and Preliminary Bioactivity Testing

**Figure 1:**
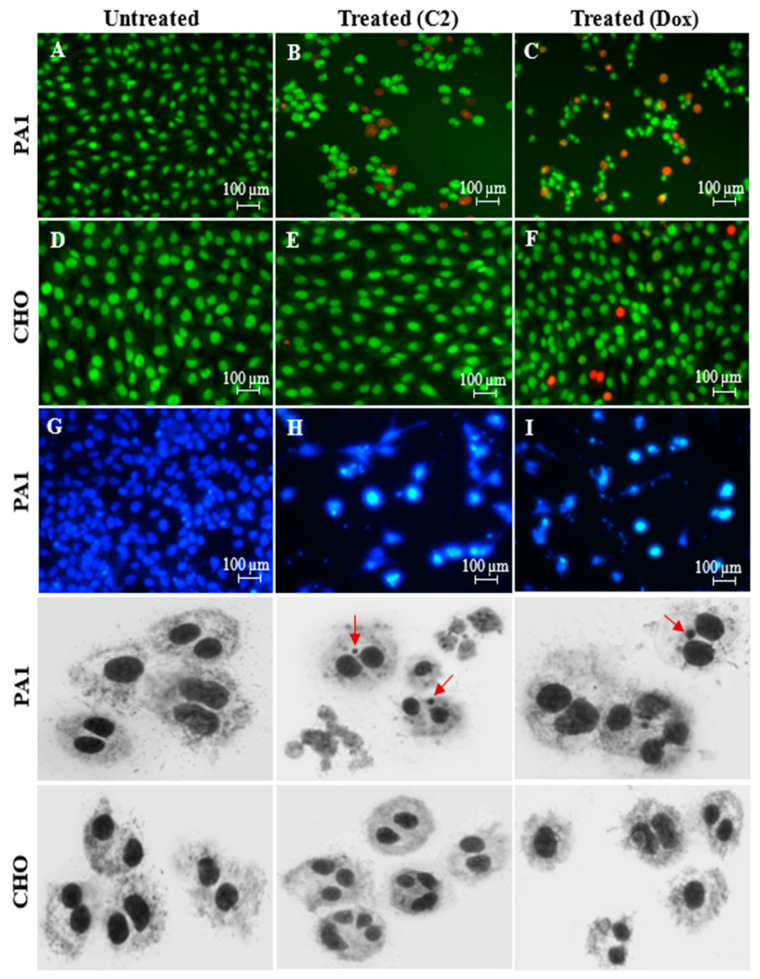
PA1 cell lines were treated with **C2** (IC_50_ 1 µg/mL (1.7 µM)) and a reference drug, doxorubicin (IC_50_ 5 µg/mL (8.6 µM)). Acridine orange/propidium iodide-stained (**A**–**F**) and DAPI-stained (**G**–**I**) cells in epifluorescent microscope view show apoptotic bodies (red), while the CHO cells were found healthy upon **C2** treatment. Micronuclei formation (red arrows) was seen for **C2**- and doxorubicin-treated PA1 cells at 20× in an Axioscope A1 Biology Microscope which is not visible for CHO, indicating **C2** is not genotoxic in non-cancerous cell lines.

We have previously isolated a fatty acid from the deep sea snail *Conus inscriptus* and the structural and chemical properties have been thoroughly investigated using Fourier transform infrared (FT-IR) spectroscopy (Jasco 4000 series, Easton, MD, USA), gas chromatography–mass spectrometry (GC-MS) (Perkin Elmer, Clarus-600 GC/MS), electrospray ionization–mass spectrometry (ESI-MS) (Shimadzu LCMS-8040 Triple Quadrupole System coupled with UHPLC (NEXERA) and Nuclear Magnetic Resonance (NMR) spectroscopy (Bruker Ultrashield 400 MHz (Avance III)) for ^1^H and ^13^C analyses, for 1D and 2D NMR, COSY, DEPT-135, HMBC and HSQC was used. Based on the spectral data, we confirm that the molecule **C2** is a bis(palmitoleic acid) ester of a glycerol and the IUPAC name of the compound is (9*Z*,9′*Z*)-3-hydroxypropane-1,2-diyl bis(hexadec-9-enoate) [3-hydroxypropane-1,2-diyl dipalmitoleate] (molecular weight of 564.48 Da) (Figure S1, Supplementary Materials). All the spectral data summarizing the chemical and structural properties of the compound have been submitted to the PubChem database with the following links: https://pubchem.ncbi.nlm.nih.gov/substance/404333224 and https://pubchem.ncbi.nlm.nih.gov/compound/14275348; accessed on 1 April 2021]. The compound, **C2,** caused cell death in PA1 cells at an IC_50_ value of 1.7 µM, which was 5 times lower than that of the reference standard doxorubicin (8.6 µM). The results are shown as AO/PI- and DAPI-stained cells with condensed nuclei (Figure 1B,H for **C2** treatment and Figure 1C,I for doxorubicin treatments, respectively). Non-cancerous CHO cells were not affected by **C2** treatment even at 60 µg (106.29 µM) (which is 60 times higher than the IC_50_ used for PA1). In addition, **C2** was not found to be genotoxic to CHO cells because the frequencies of occurrence of micronucleated cells were reported as 0.0035 ± 0.0007, 0.0045 ± 0.0007 and 0.0055 ± 0.0021 which is indicative of the safety of **C2**. At the same time, the frequencies of the occurrence of micronuclei in PA1 cells were 0.019 ± 0.0021, 0.106 ± 0.0077 and 0.098 ± 0.0042, which indicate that **C2** is genotoxic in the PA1 cell line only. All these results are given as photomicrographs in Figure 1.

### 2.2. Effects of C2 on Cell Migration, Cell Cycle Arrest, Mitochondrial Membrane Potential (MMP) Disruption and Gene/Protein Expression in PA1 Cells

**C2** was shown to inhibit migration in PA1 cells at the end of 24 h of treatment and this was compared with the control and standard-drug-treated groups. It was observed that while **C2**-treated cells were only able to migrate 1.09%, the cells in the untreated group invaded the scratched area to 86.4%. Doxorubicin-treated cells filled 2.4% of the gap area. This indicates that the compound has slightly higher anti-invasive properties than the standard.

**Figure 2:**
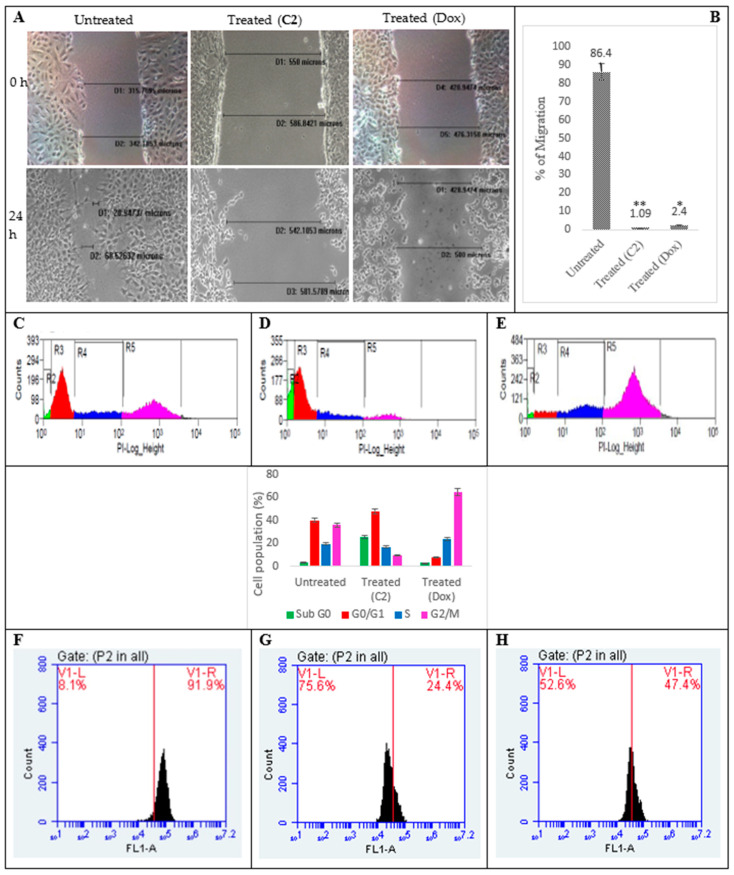
C2/Doxorubicin-treated (**A**) PA1 cell migration was inhibited (gap filling compared for 0 and 24 h and the percentages are given at 24 h: 1.09 and 2.4%, respectively) compared to their untreated controls (86.4%), showing their anti-invasive properties (**B**) [* *p* < 0.05, ** *p* ˂ 0.005]. The cells accumulated at G0/G1 phase for the **C2**-treated groups (**D**) and at G2/M for doxorubicin (**E**) compared to their untreated controls where cells were distributed across all phases (**C**) (color code: green—G0; red—G0/G1; blue—S; and pink—G2/M phase). Clear MMP depolarization was seen in **C2**-treated PA1 cells (75.6%) (**G**) from the untreated controls (8.1%) (**F**). (**E**,**H**) correspond to doxorubicin-treated positive controls.

**Figure 3:**
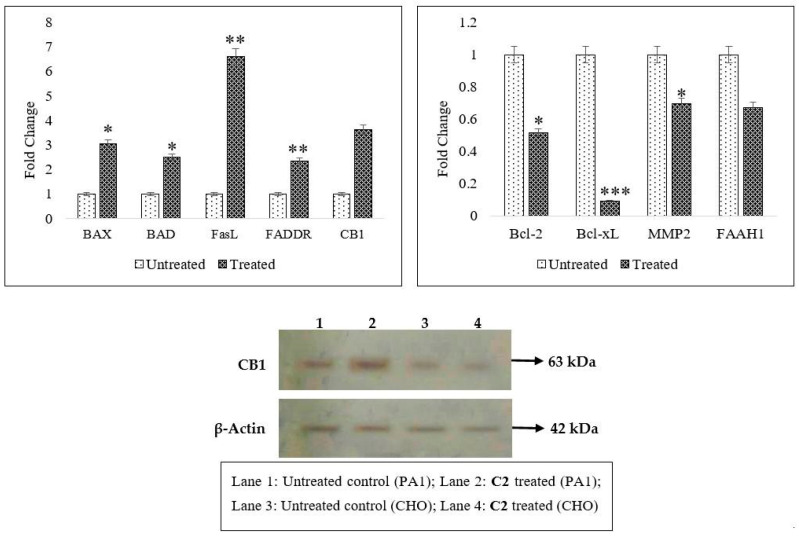
Enhanced expression of mitochondrial-associated (*BAX* and *BAD*) and death receptor (*FasL* and *FADDR*) transcripts alongside restricted antiapoptotic (*Bcl-xL, Bcl-2* and *MMP2*) mRNA transcripts (on a real-time basis), hinting at intrinsic as well as extrinsic apoptotic routes for **C2** (* *p* ˂ 0.05, ** *p* ˂ 0.005, *** *p* ˂ 0.001) in PA1 cells. In silico analysis predicted that **C2** binds to CB1 receptor as well as FAAH1, a membrane-bound enzyme (which degrades endogenous anandamide), as confirmed by molecular docking, MD simulations and RMSD results (Figure S2). CB1 expression both at gene (threefold higher) and protein levels (immunoblots are provided; β-actin served as loading control) was triggered upon treatment with **C2**. On the contrary, FAAH1 expression was 0.4 times lower than that of untreated control and this could be a reason for the maintenance of steady bioavailability of **C2** without degradation in the treated PA1 cell population.

The compound induced DNA fragmentation and arrested cell cycle at G0/G1 (48% cells) (Figure 2D), whereas for doxorubicin, we noticed the already reported G2/M arrest (Figure 2E). The untreated counterparts had cells distributed across all phases (Figure 2C). There is reported MMP depolarization and 75.6% and 52.6% of the respective cells were gated out following treatment with **C2** and Dox when compared to control. There was an altered gene expression in PA1 cells on a real-time basis and the mRNA expression levels of the apoptotic genes, BAX and BAD, increase at least 2-2.5 times (Figure 3) and there was also an increase in the FasL and FADDR mRNA levels, indicative of **C2**-mediated cell death via both intrinsic and extrinsic apoptotic pathways. BAX and BAD ratios highly responsible for the regulation of mitochondrial membrane potential (MMP) balance were also significantly altered. Conversely, mRNA transcripts of the cell survival genes (Bcl-2, Bcl-xL and matrix metalloproteinase 2 (MMP2)) were found to be 0.4–0.8-fold lower (*p* < 0.05) when compared to the untreated controls.

**C2**-induced apoptosis (both by intrinsic and extrinsic routes), micronuclei formation and altered gene expression orchestrated PA1 cell death. In silico tools were used to understand the possible targets for **C2**. The SwissTargetPrediction server (version 2014, http://old.swisstargetprediction.ch/ Accessed on 2 April 2020) predicted a list of targets, out of which, two of the targets, cannabinoid receptor type I (CB1), and a fatty acid metabolizing enzyme, fatty acid amide hydrolase type I (FAAH1), which play major, yet complicated, roles in cancer progression and invasion, were chosen for the current study (Figure S1). A series of cascading events involved in the **C2**-induced CB1 pathway will be discussed herein. To start with, expression of CB1 mRNA transcripts was three times higher and so was the protein expression in the treated cells. On the contrary, the mRNA levels of the transmembrane enzyme FAAH1 were 3.3-fold lower than those of the untreated cells and so it can be postulated that lower FAAH levels could perhaps have protected **C2** from being metabolized in the cancerous cells because FAAH1 is an endogenous CB1 ligand (anandamide)-metabolizing enzyme. This is confirmed by the sustained action of **C2** in PA1 cell death with 24 h treatment.

### 2.3. CB1 Overexpression in PA1 Cells and Acute Generation of Intracellular Ceramide (Especially C16:0 and C18:0) Pools Are Attributed to C2 Treatment

Overexpression of CB1 and its signal transduction lead to either acute accumulation or de novo synthesis of ceramide, which is a core sphingolipid class of molecules. It is evident that ceramides play pivotal roles in apoptotic signaling, cell cycle and differentiation and/or senescence. **C2**-induced overexpression of CB1 led to the accumulation of specific long-chained ceramides, C16:0 and C18:0. These two ceramides are particularly linked to apoptotic cell death in major cancer types.

**Figure 4:**
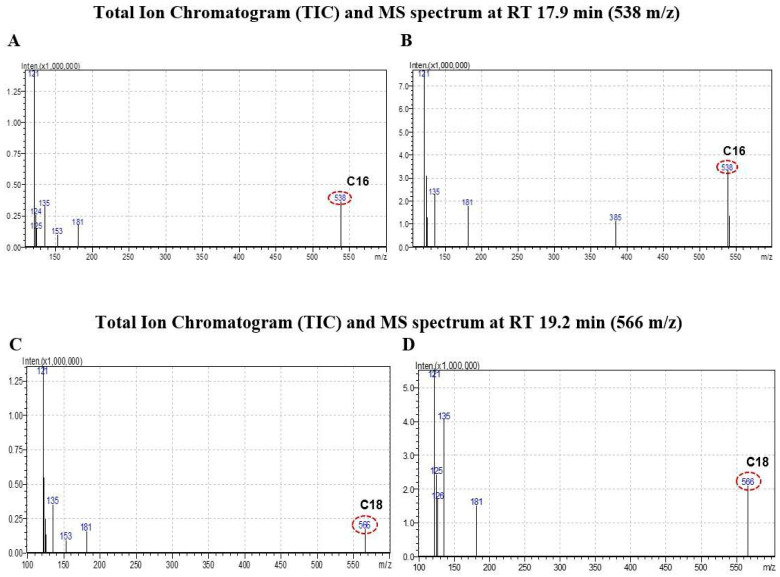
Ceramide peaks at C16:0 and C18:0 respectively were seven (**B**) and ten times (**D**) higher in the treated over control groups ((**A**) and (**C**) respectively) at the end of 16 h treatment regimen emphasizing **C2**-induced over-production of ceramides.

In the current investigation, these fatty acids were detected using LC-MS, wherein the C16:0 peak appearing at 538 *m*/*z* at 17.9 min was at least 7 times higher in the **C2**-treated PA1 cells than the uninduced cells. Similarly, the C18:0 peak appearing at 566 *m*/*z* at 19.2 min was found to be tenfold higher than in the control (Figure 4 and Figure S3). These results interestingly suggest acute accumulation of ceramides which led to the programmed cell death of PA1 cells.

### 2.4. In Vivo Monitoring of Neurotoxic Effects, Neurobehavioral Endpoints and Histopathological Examination of Brain Tissues of Freshwater Fish, Danio Rerio, Exposed to C2

Fish exposed to **C2** at different concentrations, 100, 75, 50, 25 and 12.5 mg/L (as mentioned in OECD, no:203 guideline), showed no clinical signs of mortality or behavioral anomalies and the fish remained healthy even at the highest concentration. Euthanized fish at the end of 96 h of treatment which were subjected to detailed gross examination had no observable gross lesions. Therefore, based on the acute toxicity testing, it is conclusive that LC_50_ of **C2** is not toxic as there was 0% mortality (95% confidence limits) even at the highest concentration used and that the drug is safe for further analyses that involve neurobehavioral endpoints in zebrafish models. Hence, the two highest concentrations used in the acute toxicity test were taken as Group I—high-dose **C2** (100 mg); Group II—mid-dose **C2** (75 mg); Group III—ethanol control (0.5%) and Group IV—untreated control.

**Figure 5:**
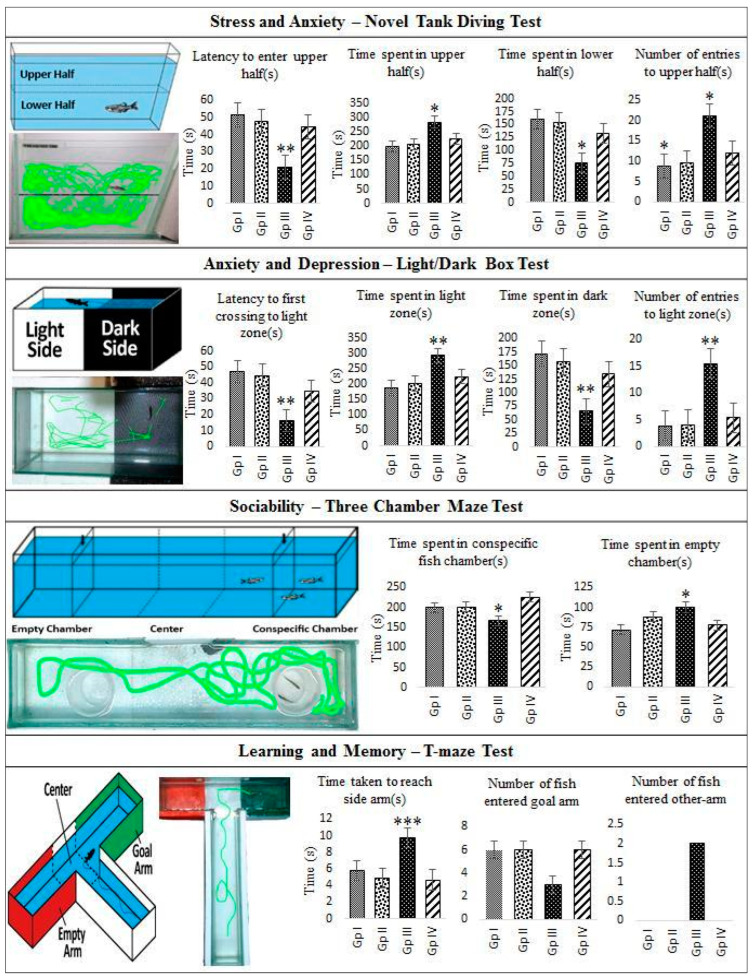
Behavioral effects of **C2** using zebrafish models: Evaluation of anxiolytic/anxiogenic properties of **C2** using novel tank diving test, light/dark box test and three-chamber maze test. All three tests revealed that both high and mid-doses are anxiolytic and that **C2** treatment does not alter neuro-related functions though **C2** is a ligand of CB1. Note that the ethanol control groups had anxiety-specific endpoints, like inhibition to diving, longer time spent in the upper half of the tank/bright zones/empty chamber. **C2** is also shown to not affect the learning and memory capabilities of the fish as seen in the T-maze test. Statistically significant differences between **C2**- and ethanol-treated groups are indicated as * *p* < 0.05, ** *p* ˂ 0.005, *** *p* ˂ 0.001. Group I—high dose **C2** (100 mg); Group II—mid-dose **C2** (75 mg); Group III—ethanol control and Group IV—untreated control. Each group contained six fish and the results are presented as average values ± SE. Panels are based on our own observations validated using MATLAB-based tracking software (Mathworks Inc., Natick, MA, USA).

**Figure 6:**
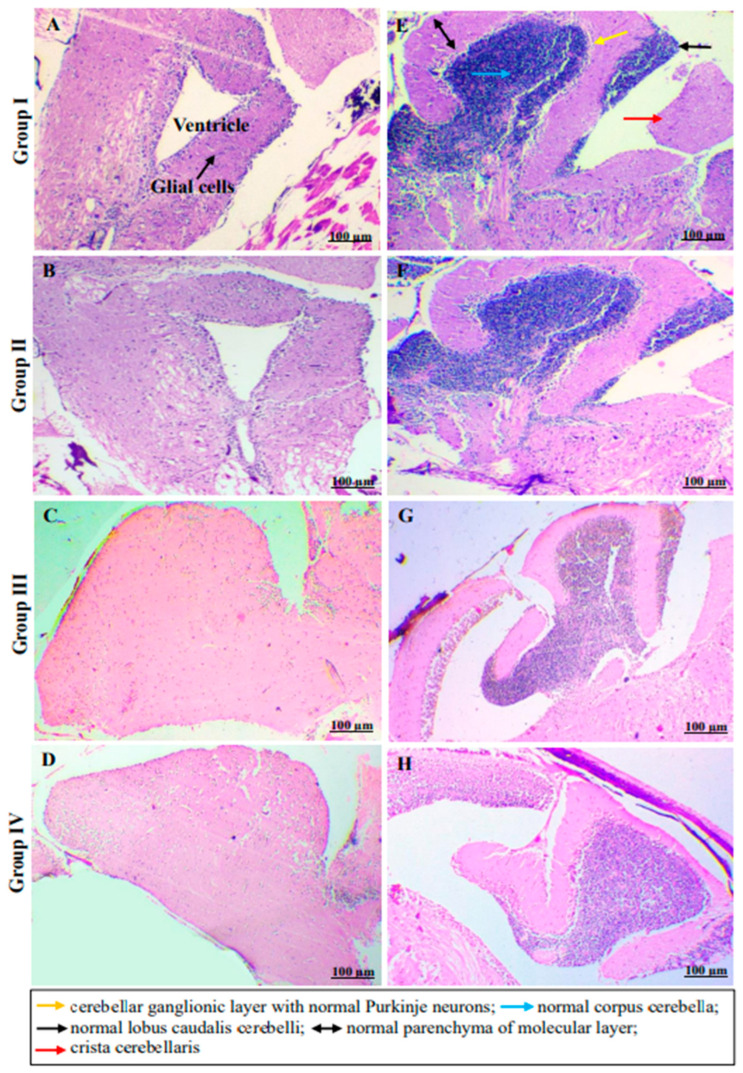
Representative hematoxylin and eosin (**H**,**E**)-stained photomicrographs showing sagittal section of hind brain tissues of zebrafish provide evidence of ventricle (**A**,**B**) and olfactory lobes (**C**,**D**) with normal architecture. The sagittal sections of cerebellum across all the groups show normal architecture (**E**–**H**) indicative of (at least) no acute brain damage upon treatments with C2 (high and mid-doses) (long term exposures need to be ascertained).

Experiments involving novel diving tanks, light/dark box or three-chamber maze tests using the drug **C2**, interestingly, did not have an anxiogenic endpoint, though it is a CB1 ligand. Any major pathway stimulated by CB1 invariably ends in a psychoactive response, however, in the current study, it is shown that the compound did not trigger a psychoactive pathway. Conversely, **C2** was indeed anxiolytic as the fish spent a considerably shorter time (205 sec ± 12.371, *p* value 0.180) with the fewest recorded entries (9.6 ± 3.262, *p* value 0.193) in the upper part of the tank compared to those treated with ethanol (284 sec ± 19.688, * *p* value 0.049 and 21 ± 2.561, * *p* value 0.011, respectively). In the light/dark box test, the numbers of entries to the light zones were significantly smaller for Groups I and II compared to the group which received positive control. A clear examination of the computer-generated traces revealed a pattern of activity, i.e., the anxiolytic **C2** induced an overt bottom dwelling/more entries to the dark chamber alongside sociability patterns similar to untreated controls. In all cases, ethanol induction led to a greater anxiety-like behavior. It also caused more entries to and more time spent in the bottom, darker zones and conspecific fish chambers, which demonstrated reduced anxiety. In any case, treated fish both at high and mid-doses behaved similarly to the untreated controls and as such there were no statistically significant differences between the high and mid-doses. Spatial memory and the rate of learning of platform location were gauged by the latency to platform discovery in fish with lower cognitive abilities in a T-maze test. The **C2**-treated fish moved to the goal arm as previously taught more quickly in comparison to the ethanol-treated ones with a statistically significant difference. This indicated retention of long-term memory with no recorded entry into the empty arm, whilst all the ethanol-treated ones invariably entered the empty arm. All the data with relevant pictures are represented in Figure 5. These results collectively suggest that **C2** treatment in zebrafish proved anxiolytic and retained sociability as well as learning and long-term memory skills. These endpoints indicate that though **C2** is a potent stimulator of the CB1 pathway, it does not produce any psychoactive response. Histopathological examination of the sagittal section of hind brain tissues of the treated and untreated groups showed no abnormalities in the ventricle nor olfactory lobes. The sagittal section of the cerebellum that showed normal architecture also indicated no acute brain damage to the fish and the representative Hematoxylin and Eosin (H and E)-stained cells are shown in Figure 6. Though histopathological investigation supports only acute exposures, long-term exposure is necessary to understand the mechanism of **C2**.

## 3. Materials and Methods

### 3.1. Materials

Eagle’s minimum essential medium (MEM), 100× antibiotic–antimycotic solution, tissue culture grade DIMETHYL SULFOXIDE (DMSO), trypsin-EDTA and 3-(4,5-dimethylthiazol-2-yl)-2,5-diphenyltetrazolium bromide (MTT) were purchased from HiMedia (Bombay, India). Tissue culture plates and flasks were procured from SPL Life Sciences (Pocheon, Korea). Fluorescent dyes used for staining the cells and nucleus, Acridine Orange/propidium iodide (AO/PI), 4,6-diamidino-2-phenylindole (DAPI) and Giemsa were from Sigma (St. Louis, MO, USA). Molecular biology grade EDTA buffer and Rhodamine 123 were purchased from Merck (Branchburg, NJ, USA). Fetal bovine serum (FBS) (Gibco, New York, NY, USA), reference standard, doxorubicin (DOX) (Adriamycin, Mol. wt: 579.98 Da, Selleckchem, Cambridge, UK), cytochalasin-B (CytoB) (Mol. wt: (479.617 g/mol; Cayman, Ann Arbor, MI, USA), proteinase K (Genei, Bengaluru, India), extra pure agarose (Lonza, Basel, Switzerland), RNAisoPlus reagent (TaKaRa, Shiga, Japan), RevertAid First Strand cDNA synthesis kit (ThermoFisher Scientific, Bedford, MA, USA) were procured from the respective sources listed in the brackets. Primary antibodies for cannabinoid receptor 1 (CB1) (Alomone labs, Jerusalem, Israel), β-actin and HRP-conjugated antirabbit IgG were procured from Santa Cruz Biotechnology (Santa Cruz, CA, USA). Organic solvents (chloroform and methanol) were of analytical grade with 98% purity and purchased from Avra Synthesis (Hyderabad, India).

### 3.2. Cell Culture, Maintenance and Assessment of Cell Viability by MTT Assay

A human ovarian teratocarcinomic (PA1) and Chinese hamster ovarian (CHO) cell line were obtained from the National Centre for Cell Science (NCCS), Pune, Govt. of India. Both cell types were grown in T-25 cm^2^ vented cap culture flasks using MEM supplemented with 10% FBS and 1% 100 × antibiotic–antimycotic solution [Streptomycin (10 mg), Penicillin (10,000 units) and Amphotericin B (25 μg)]. Cells were maintained at 37 °C, pH 7.2 in 5% CO_2_, 95% air and trypsinized twice a week; passage number was always restricted to 15.

Cell viability was assayed using a colorimetric technique that is based on the ability of live cells to convert MTT, a tetrazolium component, into purple formazan crystals by active mitochondrial dehydrogenases [19]. Both cell types were inoculated in 96-well plates at densities of 1 × 10^3^ cells in a well, a day prior to the treatment. The cells were treated with **C2** (1–60 µg after optimization) while doxorubicin (1–10 µM based on previous literature) served as a positive control. After 24 h, 100 µL (stock: 5 mg/10 mL) of MTT was added into each well and incubated at 37 °C for 3 h. The formazan crystals formed were dissolved in DMSO (100 µL) and the intensity of the color was read at 570 nm (EnSpire Multimode Plate reader, PerkinElmer, Shelton, CT, USA). From the values obtained, the percentage of viable cells was calculated as (OD of the test/OD of the control) × 100 and, from this, inhibitory concentration 50 (IC_50_) values for **C2** and doxorubicin for the cell types were calculated.

### 3.3. Assessment of PA1 Cell Death Using Nuclear Stains: Dual-Stained Acridine Orange–Propidium Iodide (AO/PI) and 4′,6-Diamidino-2-Phenylindole (DAPI) and Genotoxicity Analysis by Cytokinesis-Block Micronucleus (CBMN) Assay

Both cell types were seeded at a concentration of 1 × 10^5^ cells/well on a 6-well flat bottom tissue culture plate and added with 10% medium to attain confluence. The cells were treated with the test samples (**C2**/doxorubicin) at the respective IC_50_ and incubated for 24 h. Cells were pre washed with phosphate-buffered saline (PBS, pH 7.2) and, to it, 10 μL of AO/PI (stock: 10 μg of the respective stain/mL PBS) was added to visualize nuclear changes in an Eclipse inverted microscope (Nikon T*i* E series, Tokyo, Japan) using fluorescent filters (E_x_/E_m_: AO: 500/526 and PI: 493/636 nm). Also, the cells were washed with PBS and added with 4% paraformaldehyde for 10 min at 30 °C for fixing and treated with 0.2% Triton X-100 in PBS for 10 min at the same temperature to induce cell permeability. Thereafter, the cells were incubated with DAPI (0.5 μg/mL PBS) for 5 min to visualize apoptotic nuclei, if any, with fragmented and condensed chromatin. Cells were observed using CFI60 infinity corrected bright-field objectives at excitation/emission wavelengths of 359/461 nm.

For evaluation of genotoxic effects, both PA1 and CHO cells were plated and treated with the test samples (**C2**/doxorubicin) using the same procedures briefed above. After 24 h of drug treatment, the cells were trypsinized and a density of 5×10^3^ cells was replated into fresh 30 mm tissue culture Petri dishes, each replenished with fresh media. During culture initiation, 3 μg/mL of CytoB was added so as to inhibit cytokinesis once binucleated cells formed. After 36 h (optimized), the cells were washed with PBS and fixed with ice-cold methanol: acetic acid at 3:1 ratio (5 min) and air-dried. Cells were stained with 5% Giemsa for 15–20 min and rinsed thrice in double-distilled water, air-dried and visualized at 20× magnification in a bright field (Axioscope A1 Biology Microscope, Zeiss, Oberkochen, Germany). Details such as X and Y coordinates, number of cells with micronucleus (MN), number of MNs in the binucleated (BN) cells and cells with 1, 2, 3 and 4 nuclei were taken into consideration. The images of the cells were captured using an Isis fluorescence imaging platform, Metasystems. A total of 1000 BN cells were observed and the MN frequency was calculated as the number of MNs in BN cells to the total number of BN cells observed.

### 3.4. Inhibition of Cellular Migration (Scratch Assay), Cell Cycle, Mitochondrial Membrane Potential (∆ψ_mit_) and C2-Induced Changes in Gene and Protein Profiling

PA1 cells (1 × 10^5^ cells/well) were grown on 6-well plates to form a confluent monolayer after which gaps were created by scraping off the cells across the well using sterile 10 μL micropipette tips. Then, the cells were given regular drug treatment. Migration of the cells was monitored using an inverted microscope and captured at 0 and 24 h. The distance migrated was measured twice using Image-Pro Plus Software (Version 6.3, Media Cybernetics, Rockville, MD, USA) and the mean differences between the 0th and 24th h was calculated to show the migratory distances in the treated and non-treated groups. The same protocols listed above for seeding, plating and drug treatments were followed and the 24 h treated (**C2**/doxorubicin) cells were harvested with ice-cold PBS and re-suspended in hypotonic solution containing sodium citrate, Triton X-100, RNase and PI. Thereafter, the cells were sorted based on the fluorescence intensity of DNA-bound PI in a MoFlo XDP Cell Sorter (Beckman Coulter, Brea, CA, USA) at 488 nm, capturing 15,000 events. Percentages of live and dead cells at individual phases of the cell cycle were determined using the Summit software (version 4.3.02) that comes with the machine. To analyze MMP shifts, a cationic dye like Rhodamine 123, which exhibits membrane potential-dependent accumulation, was used. Rhodamine, when accumulated in the mitochondria of live cells, exhibits higher fluorescence than when dispersed in the cytosol of membrane-compromised cells. For this, a portion of the treated (**C2**/doxorubicin) and untreated (control) cells were washed twice with PBS and incubated with 5 μg/mL of Rhodamine 123 (Merck, New Jersey, USA) for 30 min at 37 °C in the dark. Afterward, the fluorophore in the cells was excited using an FL1A filter at 540 nm in a BD AccuriTM C6 Plus personal flow cytometer (BD Biosciences, Franklin Lakes, NJ, USA). There was a reduction in the fluorescence intensities in the treated groups (which was mostly attributed to the compromised membranes) which was calculated as a percentage in comparison with the control group (AccuriTM C6 plus analysis software).

Expression of pro- and antiapoptotic genes was studied for drug-treated cells and compared with those of untreated cells. Genes like *BAX*, *BAD*, *FasL*, *FADDR*, *Bcl-2*, *Bcl-xL*, *MMP2*, *CB1* and *FAAH1* (the last two based on in silico target prediction) were chosen for investigation. Total RNA was extracted from both the batches (treated and untreated) using RNAiso Plus reagent and the purity was checked at a 260/280 nm ratio in a NanoDrop machine (NanoDrop 1000, Thermo Scientific, Waltham, MA, USA). Two micrograms of total RNA were reverse-transcribed using a RevertAid First Strand cDNA synthesis kit according to the manufacturer’s protocol. Based on the previous literature and confirmation at https://blast.ncbi.nlm.nih.gov [Accessed on 16 September 2021], primers for all the April genes were designed (listed in Table S1). Target cDNA was mixed with SYBR Green (2X) and 5 pmol of primer for the gene of interest (5–10 pmol range was used after optimization) and the final volume was adjusted to 20 μL. The following quantitative PCR (*q*PCR) workflow was adopted for real-time gene amplification in a 7500/7500 Fast Real-Time PCR System, Applied Biosystems (Carlsbad, CA, USA): 50 °C for 2 min, 95 °C for 10 min for 40 cycles, 95 °C for 15 s and finally 60 °C for 1 min. Then, fluorescence was quantified (SYBR green: E_x_/E_m_ 497/520 nm). The mRNA expression levels of all the genes were normalized with the endogenous control, beta-actin (β-actin) and the data analysis was carried out in Relative Quantification Manager software (QM, Version 1.2; [Accessed on 2 October 2022] by the comparative threshold (Ct) method. Fold changes for each of the mRNA transcripts were calculated as 2^−ΔΔCT^ [20] and the averaged values were statistically validated based on the standard errors (SEs).

For protein expression studies, the total protein content of the PA1 and CHO cells after 24 h of drug treatment (**C2**) was extracted using RIPA buffer that contained 1X protease inhibitor cocktail (25 mM Tris (pH 7.4), 150 NaCl, 1% NP-40, 0.5% sodium deoxycholate and 0.1% SDS) and then quantified by BCA assay. Around 20 µg of the total protein was separated in 10% SDS-PAGE and blotted onto a nitrocellulose membrane using semi-dry blot apparatus. The membranes were blocked for 1 hour in 5% skim milk and then washed thrice using PBST (PBS with 0.1% Tween 20) followed by incubation with primary antibodies against CB1 and β-actin overnight at 4 °C with 1% skim milk. After washing, the blots were incubated with HRP-conjugated antirabbit IgG for 1 h at ambient temperature and the proteins were detected qualitatively using a Western Blotting Detection Kit (ECL, Amersham BioSciences, Slough, Buckinghamshire, UK).

### 3.5. Analysis of Ceramide Accumulation in Response to C2 Treatment Using Liquid Chromatography–Mass Spectrometry (LC-MS)

At the end of the 16 h **C2** treatment regimen, total lipid was extracted from the cells using methanol/chloroform (1:2). For this, PA1 cells were grown in 60 mm tissue culture Petri dishes and harvested at 16 h (time optimized) after drug treatment by removing the culture medium and washing the adherent cells with 1 mL PBS. A volume of 2 mL methanol was added to each of the Petri dishes (treated and untreated control) and the cells were scraped and transferred into a 15 mL polytetrafluoroethylene capped borosilicate glass tube. Cells were sonicated for 5 min and 4 mL of chloroform was added to the cells and the suspension was vortexed for 5 s followed by another 30 min of sonication. Lysates were spun for 5 min at 3000 rpm. The lower chloroform layer was transferred to a new tube and kept on ice, whilst the upper methanolic and the middle proteinaceous layers were re-extracted using 2 mL of methanol and 4 mL of chloroform. The contents were vortexed, sonicated and spun as indicated previously. For a second time, the lower layer was removed and the collected chloroform layers were pooled, kept on ice and dried under low-nitrogen gas at room temperature. Qualitative analysis of the lipids was performed with LC-MS (Shimadzu, Kyoto, Japan) with a Nexera X2 as frontend. Samples were used as neat injection in the HPLC column and the samples were diluted to 10,000 times to show differences between C16:0 and C18:0 peaks as well as to distinguish from other ions in the LC-MS. The protocol suggested by Huang et al., 2016 [21] was used as a reference for the chromatographic conditions (Column parameters: Agilent: Zorbax RRHD Eclipse Plus C18 (100 mm, 3 mm ID* 1.8 µ) (IICMS/LCC-229); mobile phase: A: CH_3_OH/H_2_O/HCOOH (60:40:0.2, *v*/*v*/*v*) and B: CH_3_OH/CH_3_CHOHCH_3_/HCOOH (60:40:0.2, *v*/*v*/*v*), both containing 10 mM NH4OAc; flow rate: 0.4 mL/min; elution mode: gradient: 0.01/0, 3/10, 3.5/10, 5/40, 5.3/55, 8/60, 8.5/80, 10.5/80, 11/90, 19/90, 22/100, 25/100, 26/0, 30/stop; column oven temperature: 40 °C; run time: 30 min; wavelength: 254 nm; LCMS parameters: interface: ESI; interface voltage: 4.5 kV; polarity: positive and negative; nebulizing gas flow: 2 L/min; drying gas flow: 15 L/min; DL and HB temperature: 250 °C and 400 °C).

### 3.6. Effect of C2 on the Neurobehavioral Properties in Adult Zebrafish, Danio Rerio, Models

#### 3.6.1. Housing and Acclimatization

The study was carried out at Whizbang Bioresearch Private Limited, Chennai, Tamil Nadu, 600077 following OECD guideline No. 203, 17th July 1992. Approximately 70 fish were housed in a single home tank (40 L capacity) and uniformly allowed a 12-day acclimatization period. The aquarium was filled with dechlorinated purified water, pH: 6.8–7.8, and 1/3rd of the water was changed every three days and the temperature was maintained at 26 ± 1 °C. The subjects were provided with an alternating photoperiod of 12 h artificial light (ceiling-mounted fluorescent light tubes) and darkness and fed with commercial food flakes. Feeding was stopped 24 h prior to the commencement of the dosing.

#### 3.6.2. Acute Toxicity Test

After the acclimatization period, fishes were transferred to the test tank and randomized into six groups, each consisting of ten fish (male: female equal ratio). All the tanks were filled with the required volume of aquarium habitat water and provided with an aerator. Physico-chemical parameters like pH, temperature, dissolved oxygen and light were maintained at optimum levels throughout the test period. The total volume of exposure medium was 5 L per tank (not exceeding maximum load of 1g of fish per L). For the groups I, II, III, IV and V, the subjects were treated with different concentrations of **C2**: 100 mg/L, 75 mg/L, 50 mg/L, 25 mg/L and 12.5 mg/L, respectively, and group VI served as control (no treatment). The experimental fish were observed for treatment-related lethal and sub-lethal effects at 0, 6, 24, 48, 72 and 96 h. Mortality and clinical signs/behavioral abnormalities were observed and recorded throughout the test (96 h).

#### 3.6.3. Stress and Anxiety—Novel Tank Diving Test

After 96 h of exposure to **C2**, zebrafish (*n* = 6 randomly selected from each group) were placed individually in a trapezoidal tank (15.2 cm height × 27.9 cm top × 22.5 cm bottom × 7.1 cm width) filled with 1.5 L of aquarium habitat water. The tank was marked horizontally outside with a dotted line through the middle making two equal halves. Once gently transferred to the novel tank, the behavior of the fish was recorded for 6 min, both manually and using a video camera. After 6 min, fish were removed from the novel tank and placed in their respective aquarium tanks for further experimentation. Video footage was replayed thrice and scored manually by a single reviewer to obtain consistent results and validated using MATLAB-based tracking software. Behavioral endpoints of this study are latency to enter the upper half (s), time spent in the upper half (s), time spent in the lower half (s) and number of entries to the upper half [22].

#### 3.6.4. Anxiety and Depression—Light/Dark Test

The test apparatus consisted of a half-black, half-white tank (15.2 cm height × 10 cm depth × 45 cm width) with a 5 cm central area bound by sliding doors, which was used as an initial test chamber and had a 10 cm water column. The fish (*n* = 6 in each group) were individually introduced in the central area for 60 s for acclimation. The sliding doors were then removed, allowing the fish to freely explore the apparatus, and they were filmed for 6 min. The videos were then analyzed using MATLAB-based tracking software for the following parameters: time spent in light zone (s), time spent in dark zone (s), latency to first crossing to light zone (s) and number of entries to light zone [23].

#### 3.6.5. Sociability—Three Chamber Maze Test

This was carried out in a three-chamber maze (total dimensions: 30.5 cm × 15.2 cm × 15.2 cm) and the tank was divided into three chambers by placing two transparent glass dividers with sliding doors. The maze was filled up to a depth of 10 cm with aquarium habitat water. Prior to testing, there was a habituation phase of 5 min in which the experimental fish was allowed to acclimate and explore the maze. Before the test session, a conspecific fish was placed within a transparent beaker in the middle of the right arm and an empty transparent beaker was placed in the middle of the other arm. Then, zebrafish (n = 6 in each group) were individually transferred into the central zone of the maze, in which the drop-in doors were closed. After 30 s (to reduce transfer/handling stress), both the sliding doors were simultaneously lifted and the fish were allowed to swim freely and explore the maze for 6 min. The fish moved to the left/right arm (empty arm/conspecific fish arm) according to their preference. Fish behavior was scored manually by the observer and also by video recording. The endpoints are time spent in the conspecific fish chamber (s) and time spent in the empty chamber (s) [24].

#### 3.6.6. Learning and Memory—T-Maze Test

The T-maze consisted of a long central arm (50 × 10 × 10 (in cm)) and two short side arms (20 × 10 × 10 (in cm)) which was filled up to a depth of 10 cm with aquarium habitat water (28 °C). A glass door was used to hold the fish at the start point for the first 30 s to reduce the handling stress. Red and green plastic sleeves were constructed on the side arms of the T-maze. The maze was surrounded by an opaque white board to prevent undesired visual cues.

Training session: Fish were trained to reach the goal arm (green sleeve) and once the overnight fasted fish exited the starting chamber, it could swim to either of the side arms. When the fish successfully entered the goal arm and found the food reward, it was given 30 s to eat the food, after which it was returned to the holding tank. When it entered the wrong arm (red sleeve), it was taken back to the starting chamber and trained again. Training sessions were held for 5 consecutive days.

Test session: The behavior of the fish was tested on the 13th day after training sessions in order to analyze the long-term memory. The test session followed the same procedure as that of the training session but without the reward. Once the fish exited the start chamber, it was observed and recorded for a period of 6 min. The endpoints are latency to reach either side arm (s), selection of goal arm or non-preferred arm [25].

#### 3.6.7. Histopathological Examination of Brain Tissues

After all the tests were complete, the fish were euthanized in 500 mg/L tricaine methanesulfonate (Sigma–Aldrich, MO, USA) and two fish per group were randomly selected and immediately dissected to isolate the brain tissue sample. The samples were fixed in 10% buffered formalin and processed in alcohol and xylene and impregnated with paraffin. Processed tissues were embedded in a paraffin block, sectioned into 5 µ pieces and stained with hematoxylin and eosin (H&E) for histopathology examination.

### 3.7. Statistical Analyses

All the experiments were carried out thrice and the mean values are given with standard errors (mean values ± SE). Student’s *t*-test and one-way ANOVA were performed wherever required and significance was measured at three probability values (*p* values): * *p* < 0.05, ** *p* < 0.005, *** *p* < 0.001. All the results were analyzed using the software of the current release version as stated in the individual methodology sections.

## 4. Discussion

Cancers diagnosed in organs related to the female reproductive system account for one out of six cancers in females [26,27], with uterine and ovarian cancers topping the ten commonest malignancies (Cancer Research, London, UK, 2014). Estimates from global country profiles on the burden of cancer released by the WHO in 2020 indicate that there are 313,959 new cases and 207,252 deaths worldwide and 4.2–4.8% mortality rates in India alone in 2020 (reports from Global Cancer Observatory, 2020). Despite all the challenges, there have been a few biomarkers both at genetic (BRCA1 [28], Satellite 2 DNA [29], RASSF1A [30]) and protein levels (Cancer Antigen 125 [31], haptoglobin [32]) and others with lesser specificities. Currently there are 24 drugs administered singly and 7 in combination, however, there is a big list of side effects on the NIH-NCI’s page of drugs approved for specific types of cancer. Within this setting, however, the important roles played by the endocannabinoid system (ECS) in various human reproductive organs, such as the uterus, ovary, fallopian tubes and cervix, have been understood for the past two decades [33,34]. The ECS comprises prototype neuroactive molecules (anandamide), cannabinoid-type receptors (CB1, CB2, GPR55) and enzymes (FAAH1 and monoacyl-glycerol lipase (MAGL)) and there is a tight link to a wide range of cancers, including, but not limited to, those of lung, glioma, pancreas, oral and embryonal origin [35,36,37]. Among these, ovarian cancers (OCs) have also been focused on by a few researchers and clinicians [38,39], but not to the extent in other cancers. Perturbators of CB1 and FAAH1-linked pathways in gynecological cancers are mainly studied using endo/phytocannabinoid/cannabidiol ligands like ∆9-THC, WIN55, 212-2, anandamide and (+) methAEA and also HU-210 in recent years [40,41]. But there is no report on the exploration of natural compounds as CB1/FAAH1 modulators although even the slightest dysregulation of these targets could be one of the starting points for drug discoveries. Therefore, the present work investigates the role of a natural compound from a marine snail, *Conus inscriptus*, in inhibiting ovarian cancer cells with special reference to the CB1 pathway.

The results of the present investigation revealed that **C2** killed PA1 cells and it was also understood that the compound is non-genotoxic to non-cancerous cells. Even at a concentration 60 times higher (106.29 µM) than that used for PA1 cells (1.7 µM), there was no micronucleus formation in normal cells. Modulation of the cell cycle, especially in ovarian cancer cell types, has been appraised as one of the treatment strategies to prevent the cells from replicating. Drugs currently in Phase II and III clinical trials, adavosertib and adavosertib with gemcitabine in platinum-resistant OC, inhibited G2-M transition [42,43], whereas in the current study, there was a G0/G1 arrest, which resulted in total diminution of S and G2/M peaks to cause DNA laddering. Interestingly, WIN 55, 212-2 (a synthetic ligand of CB1) treatment in glioma cells, and URB597, a FAAH1 inhibitor, also arrested at G0/G1 only [35], suggesting a link between CB1 stimulation–FAAH1 inhibition and G0/G1 arrest, which is to be investigated. **C2**-dependent inhibition of PA1 invasion can be well correlated with a reduced expression of MMP2 (* *p* = 0.0512), which has a key role in metastasis.

**C2**-triggered events, including upregulation of death promoters, BAX-BAD, and downregulation of Bcl-xL and Bcl-2 survival genes that led to mitochondrial dysfunction, are revealed in the present investigation. While these events are turned on, Fas/FasL-mediated death signaling is also induced, suggesting a convergence of internal and external apoptotic routes. Gene expression studies exposed that there was a profound increase in these death factors (more than 3-fold). As indicated by computational analysis, **C2** does bind to CB1 which correlates with real-time mRNA amplification and concomitant protein expression of CB1. In general, it is known that in many cancers, with ovarian cancer of particular note, there is a moderate to strong expression of CB1 receptor [33,38,44] as encountered in the present study. In addition to this, there is another surge in CB1 expression upon treatment with agonists [45] and very fascinatingly it is also reported that agonist treatment sometimes spares non-cancerous cells, protecting them from CB1-induced apoptosis, and decreases CB1 expression [46], a phenomenon still poorly known.

Exposure of cells to any stimulus that increases intracellular ceramide levels will evoke changes in the cell to determine cell fate. In this context, it is shown that CB1 activation is coupled to the production of a second messenger, ceramide, by two pathways: i) sphingomyelin hydrolysis leading to acute increases in ceramide levels and ii) sustained de novo intracellular ceramide synthesis. In the first case, sphingomyelin hydrolysis leads to enhanced ceramide levels within 15 min upon CB1 stimulation to at least twofold more than without stimulation [47,48]. In the second case, CB1 activation evokes the generation of a sustained ceramide pool which occurs as a second peak that starts after 2–3 days of treatment, reaching a fourfold increase on the 5th day [49,50]. In the current investigation, in PA1 cells after 16 h, there was a 7-10-fold greater ceramide level than in the untreated control. However, as shown in the literature, we did not check the ceramide levels after 15 min of treatment but certainly the cells did not survive for a second ceramide peak at the end of 3 days, suggesting intracellular accumulation of a second ceramide peak. Sphingomyelin hydrolysis and a sudden surge in ceramide levels are only linked to the execution phase of apoptosis. Specific ceramides like C16:0 and C18:0 were studied because only sudden accumulation of long-chained ceramides caused apoptosis [51,52] and shorter ones, less than C10:0, did not [53]. In addition to this, several studies [54,55,56,57] have highlighted the purpose of Fas activation through the engagement of FasR which induced a 3–8-fold increase in ceramide levels within 12-24 hours which is consistent with the present finding. Not only that, ceramides are also known to invariably arrest cells at G0/G1 in a few cancers [58,59]. Though **C2** treatment-induced CB1 stimulation leading to cell death can be directly linked, the accessory roles played by upregulated FasL and FasR must also be considered if the expression of these genes is ceramide-independent.

Alongside discovering CB1 agonists, equal importance has been given to investigating FAAH inhibitors because FAAH is a catabolic enzyme for endo- and exogenous cannabinoids. By blocking this enzyme, the levels of CB ligands which primarily modulate the ECS pathway can be maintained [60,61]. Based on the in silico analysis, it was found that C2 also binds to FAAH1 with a favorable molecular docking score of −4.91 kcal/mol. This was further strengthened by molecular dynamic simulations which suggested only minor fluctuations in FAAH1-C2 bound forms through a 50-nanosecond run (previously referenced). Taken together, from the results of in silico and C2-induced inhibited expression of FAAH (0.4-fold), it may be postulated that the compound may be an inhibitor of FAAH which might have contributed to the sustained activity of **C2** even at a 1.7 µM concentration without being degraded.

Regardless of the anticancer property of **C2**, clinical application may often be limited if it has psychoactive properties, because almost 90% of CB1 agonists possess psychotropic properties, with the exception of cannabidiol. Since **C2** is a natural compound and this is the first study on its action on CB1 and as an anticancer agent, it is essential to appraise its neurobehavioral aspects. It was proved, using zebrafish models, that **C2** did not alter the neurophysiological aspects of fish nor did it induce anxiety or cognitive/learning/memory/locomotor impairment in accordance with OECD-approved guidelines. Hence, this study proves that **C2** is a non-psychotropic, safe CB1 agonist–FAAH1 blocker that also possesses anticancer properties in PA1 cell lines by elevating acute cellular ceramide levels.

## 5. Conclusions

**C2** is a brown-colored semi solid viscous compound from *Conus inscriptus*, a marine snail (yield to dried snail biomass: 160 mg/500 g). The compound is a proven apoptotic inducer and an inhibitor of G0/G1 transition. The compound is a clear controller of intrinsic (BAX, BAD, Bcl-2, Bcl-xL and MMP2) and extrinsic (FasL and FADDR) apoptotic markers at gene levels and an inducer of CB1 at both mRNA and protein levels. The compound reduced FAAH1 mRNA transcripts within 24 h of treatment. This in turn could have lowered the action of the catabolic enzyme FAAH1 which must have had a role in the continued activity of **C2** at such a reduced concentration (1.7 µM). This marine compound is a potent CB1 transducer to instantly cause an intracellular ceramide surge (seven- and tenfold for C16:0 and C18:0, respectively).

Despite being a CB1 agonist, **C2** treatments on zebrafish indicated that the compound is non-toxic even at the highest dose specified by OCED guidelines alongside not affecting neuronal behavior/brain function in those fish. In addition, **C2** did not cause cognitive/learning/memory/locomotor impairment. From this study, it could be seen that fatty-acid-type molecules from natural sources possessing structural similarities to endogenous cannabinoids are capable of inducing intracellular ceramide accumulation. Thus, this molecule can be explored for ovarian cancers as a potent CB1 agonist and FAAH1 inhibitor.

## Data Availability

The data generated in this study are available upon request from the corresponding author.

## Supplementary Materials

The following supporting information can be downloaded at: https://www.mdpi.com/article/10.3390/molecules29081737/s1, Figure S1: Structure of C2 with molecular details (A) and Select targets used for the present study [SwissTargetPrediction server (version 2014) http://old.swisstargetprediction.ch/ (accessed on 1 April 2021)] (B); Figure S2. Molecular docking analysis (A, B), Root Mean Square Deviations (RMSD) (C, D) and Radius of gyrations (E, F) for C2-target [CB1 and FAAH1] complex [color coding: purple—CB1; red—CB1 with C2; blue—FAAH1; black—FAAH1 with C2]; Figure S3. Total Ion Chromatogram at RT 17.9 min (538 *m*/*z*) indicate C:16 ceramides [Above: control; Below: treated]; Table S1. Primer chart (forward and reverse) used in *q*-PCR studies; Video S1: Video footage for all neurobehavioral parameters recorded and validated using MATLAB-based tracking software [*ToxTrac*] which is an automated executable software for image-based tracking that can simultaneously handle several organisms monitored in a laboratory environment. We compared the performance of *ToxTrac* with manual scoring too. Videos available upon request to the corresponding author.
