# Supplementary material for: A Novel Non-Psychoactive Fatty Acid from a Marine Snail, Conus inscriptus, Signals Cannabinoid Receptor 1 (CB1) to Accumulate Apoptotic C16:0 and C18:0 Ceramides in Teratocarcinoma Cell Line PA1
Source: Molecules. 2024 Apr 11;29(8):1737. doi: 10.3390/molecules29081737 (PMC11052367; doi:10.3390/molecules29081737)
Supplement: Supplementary file 1 [file molecules-29-01737-s001.zip › molecules-2793370-supplementary.pdf]

A

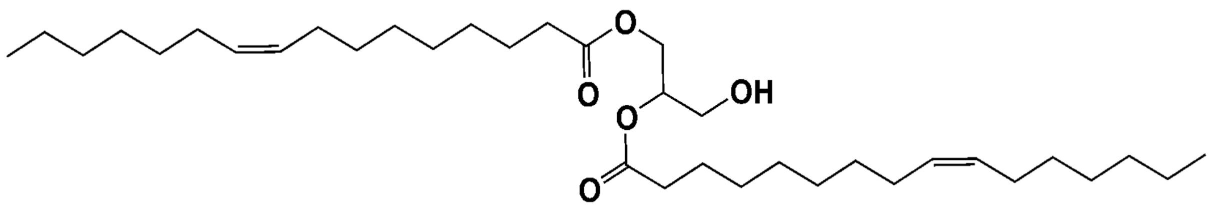

(9Z,9'Z)-3-hydroxypropane-1,2-diyl bis(hexadec-9-enoate)
Chemical Formula: $C_{35}H_{64}O_5$
Exact Mass: 564.48
Molecular Weight: 564.88

Target	Uniprot ID	Gene code	ChEMBL ID	Probability	# sim. cmpds (3D / 2D)	Target Class
Protein kinase C gamma type *(by homology)*	P05129	PRKCG	CHEMBL2938		0 / 213	Ser_Thr Kinase
Protein kinase C beta type *(by homology)*	P05771	PRKCB	CHEMBL3045		0 / 213	Ser_Thr Kinase
Protein kinase C alpha type	P17252	PRKCA	CHEMBL299		0 / 213	Ser_Thr Kinase
Protein kinase C theta type	Q04759	PRKCQ	CHEMBL3920		0 / 217	Ser_Thr Kinase
Protein kinase C delta type regulatory subunit	Q05655	PRKCD	CHEMBL2996		0 / 217	Ser_Thr Kinase
Fatty-acid amide hydrolase 1 *(by homology)*	O00519	FAAH	CHEMBL2243		0 / 23	Enzyme
Cannabinoid receptor 1 *(by homology)*	P21554	CNR1	CHEMBL218		1 / 33	Membrane receptor
Cannabinoid receptor 2	P34972	CNR2	CHEMBL253		1 / 19	Membrane receptor
Monoglyceride lipase *(by homology)*	Q99685	MGLL	CHEMBL4191		0 / 12	Enzyme
Protein kinase C eta type *(by homology)*	P24723	PRKCH	CHEMBL3616		0 / 9	Ser_Thr Kinase
Protein kinase C epsilon type	Q02156	PRKCE	CHEMBL3582		0 / 9	Ser_Thr Kinase
Uncharacterized protein *(by homology)*	H0YJX3				0 / 2	Ser_Thr Kinase
Transient receptor potential cation channel subfamily V member 1 *(by homology)*	Q8NER1	TRPV1	CHEMBL4794		0 / 3	Ion channel
Microtubule-associated protein tau	P10636	MAPT	CHEMBL1293224		0 / 27	Unclassified
Prostaglandin G/H synthase 2	P35354	PTGS2	CHEMBL230		0 / 16	Enzyme

B

Figure S1. Structure of **C2** with molecular details (**A**) and Select targets used for the present study [SwissTargetPrediction server (version 2014) http://old.swisstargetprediction.ch/] (**B**).

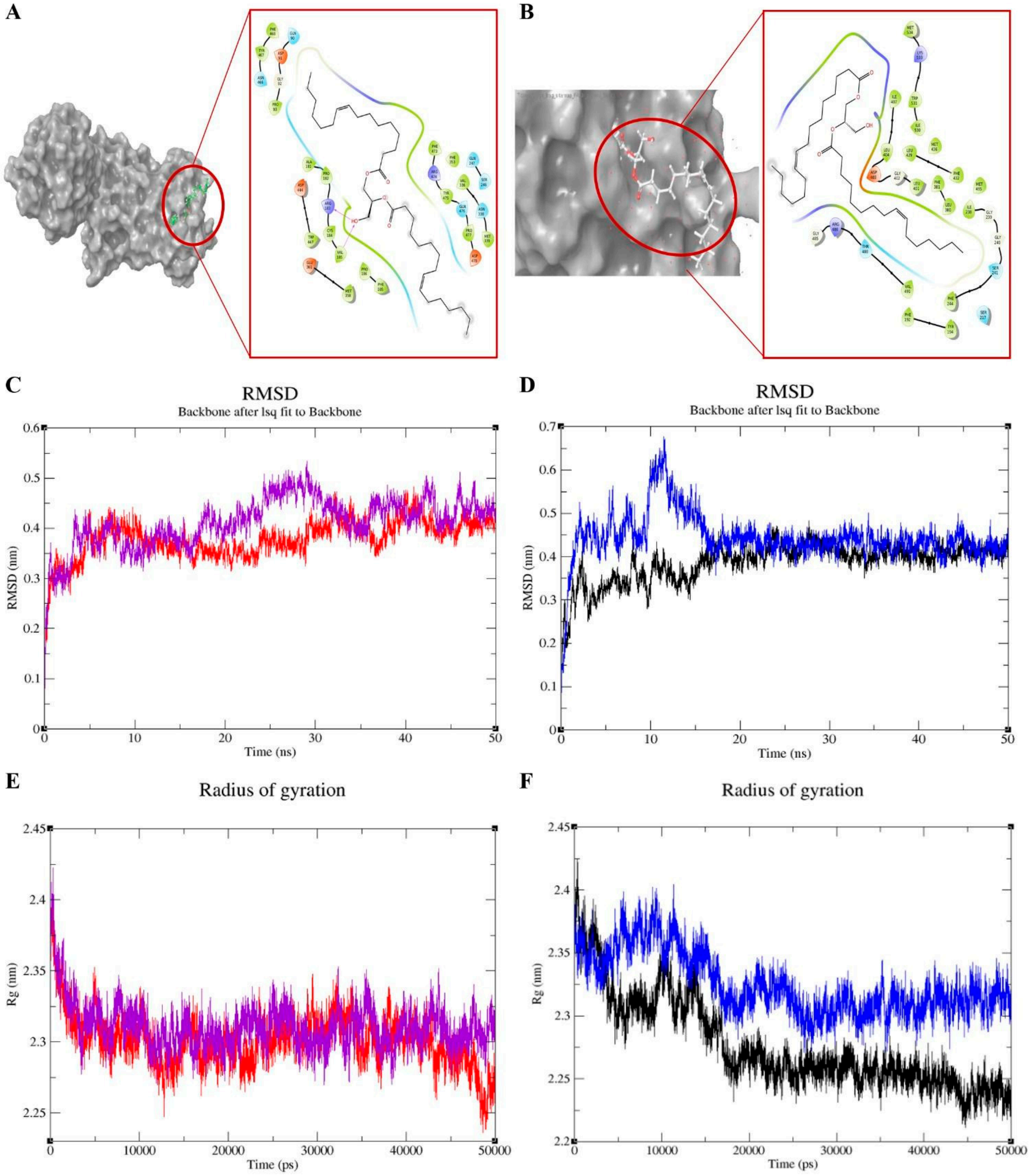

Figure S2. Molecular docking analysis (**A, B**), Root Mean Square Deviations (RMSD) (**C, D**) and Radius of gyrations (**E, F**) for **C2**-target [CB1 and FAAH1] complex [color coding: purple - CB1; red - CB1 with **C2**; blue – FAAH1; black - FAAH1 with **C2**].

Figure S3. Total Ion Chromatogram at RT 17.9 min (538 m/z) indicate C:16 ceramides [Above: control; Below: treated]

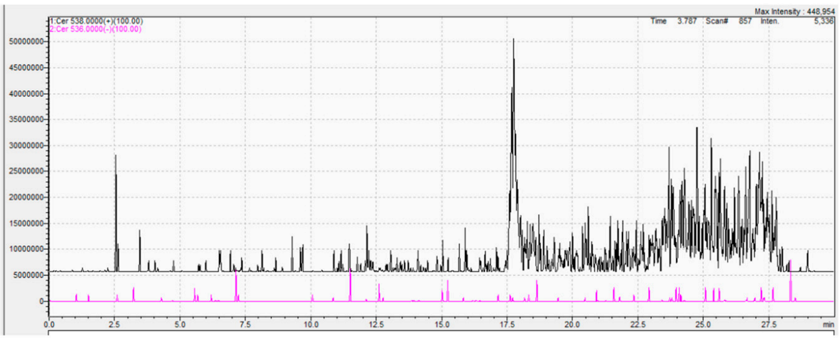

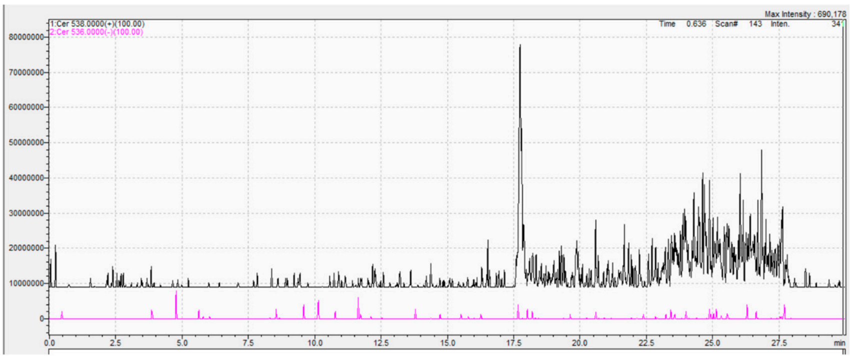

Total Ion Chromatogram at RT 19.2 min (566 m/z) indicate C:18 ceramides [Above: control; Below: treated]

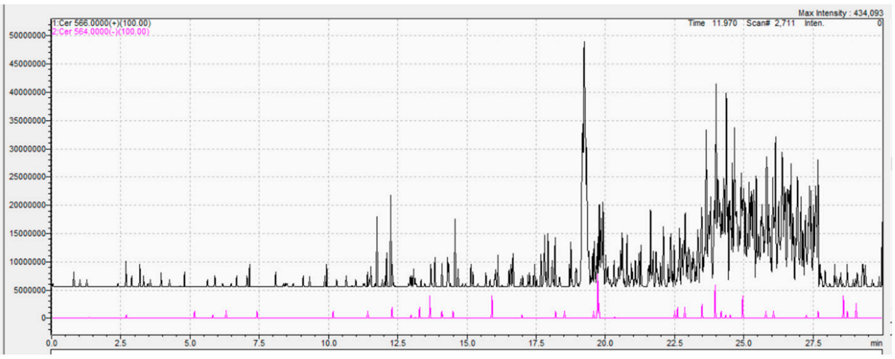

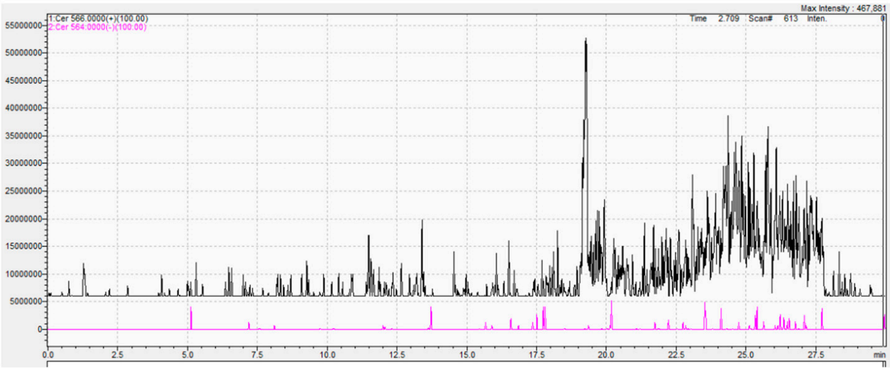

Gene	Orientation	Primer sequence 5'- 3'	Reference / GenBank Accession Number
β-Actin	F	GGACTTCGAGGAAGAGATGG	NM_001101.5
	R	AGCACTGTGTTGGCGTACAG	
BAX	F	GCTGGACATTGGACTTCCTC	Elumalai et al., 2012
	R	CTCAGCCCATCTTCTTCCAG	
BAD	F	CCTCAGGCCTATGCAAAAAG	
	R	AAACCCAAAACTTCCGATGG	
FasL	F	CCATGTGAAGAGGGAGAAGC	
	R	AAGACAGTCCCCCTTGAGGT	
Bcl-xL	F	GGCTGGGATACTTTTGTGGA	
	R	AAGAGTGAGCCCAGCAGAAC	
Bcl-2	F	ATTGGGAAGTTCAAATCAGC	
	R	TGCATTCTTGGACGAGGG	
FADDR	F	AGATGAACCTGGTGGATGAC	
	R	AGGACGCTTCGGAGGTAGAT	
MMP2	F	TTTCCATTCCGCTTCCAGGGCAC	Zhan et al., 2012
	R	TCGCACACCACATCTTTCCGTCACT	
CB1	F	AGGAGTAAGGACCTGCGACA	U73304.1
	R	TCTTGACCGTGCTCTTGATG	
FAAH1	F	GGCCACACCTTCCTACAGAA	NM_001441.3
	R	GTTTTGCGGTACACCTCGAT	

Table S1. Primer chart (forward and reverse) used in *q*-PCR studies
